# Loudness perception deficits during altered and absent auditory feedback in Parkinson’s disease

**DOI:** 10.3389/fnhum.2025.1521748

**Published:** 2025-03-28

**Authors:** Dona Anita Senthinathan, Scott G. Adams, Allyson D. Page, Mandar Jog

**Affiliations:** ^1^Department of Speech-Language Pathology, SUNY Buffalo State University, Buffalo, NY, United States; ^2^Department of Health and Rehabilitation Sciences, Western University, London, ON, Canada; ^3^School of Communication Sciences and Disorders, Western University, London, ON, Canada; ^4^Department of Clinical Neurological Sciences, Western University, London, ON, Canada

**Keywords:** Parkinson’s disease, speech perception, speech loudness, altered auditory feedback, masking noise

## Abstract

Patients with Parkinson’s disease (PD) present with speech difficulties including abnormal speech intensity regulation. It is possible that the neural circuitry in speech may be unique and more complex compared to the other major motor symptoms. The current study aimed to provide a better understanding of the sensorimotor integration and loudness perception deficits in PD using an altered intensity feedback (AIF) paradigm. Twenty-six participants with PD and 26 neurologically healthy control participants completed a magnitude production task (normal loudness, 2× louder, 4× louder, and max loudness) while being presented with AIF and background noise. The task was repeated in complete masking noise and loudness perception ratings were obtained in all conditions (no noise and background noise). Results suggest that unlike previous studies in other sensorimotor domains, individuals with PD display a reduced reliance on auditory sensory feedback such that during a speech magnitude production task, their perception of those productions may rely less on the auditory sensory feedback being received. Loudness perception results in the absence of auditory feedback suggest a modulating effect of sensory feedback on somatosensation or sense of effort in PD.

## Introduction

Dopaminergic cell loss in the substantia nigra pars compacta in individuals with Parkinson’s disease (IWPD) is associated with disruption of the basal ganglia-thalamocortical motor circuit (Brug et al., 2015). Several previous studies have suggested sensorimotor integration deficits such that there is an overreliance on sensory information and impaired movement accuracy in the absence of sensory feedback (Almeida et al., 2005; Bronstein et al., 1990; Klockgether and Dichgans, 1994; Rinalduzzi et al., 2015). For example, a study by Teulings et al. (2002) found overreliance of visual feedback by IWPD in a writing task contrary to controls who updated their original prediction using the manipulated visual feedback and made corrections to their handwriting movements in the expected/opposite direction to the perturbed error (Teulings et al., 2002). IWPD present with hypokinetic dysarthria due to the hypokinetic symptoms of the speech system (reduced force and amplitude of movement). Hypophonia or low speech intensity has been found to be the most common speech symptom experienced by IWPD, across age and disease duration (Adams and Dykstra, 2009; Darley et al., 1969; Duffy, 2013; Logemann et al., 1978; Wertheimer et al., 2014). The relationship between the major motor symptoms of Parkinson’s disease (PD) and speech symptoms are unclear, suggesting that basal ganglia involvement in speech may be unique and more complex.

The degree to which auditory sensory feedback is utilized to regulate speech intensity is unclear. The specific process and neural mechanism by which auditory speech intensity information is processed may be better understood using error correction tasks. The current study aimed to provide a better understanding of the sensorimotor integration [process by which peripheral sensory pathways convey information to cortical motor pathways and this information is then integrated by the central nervous system in order to complete motor program execution, Abbruzzese and Berardelli (2003)] and potential loudness perception deficits associated with PD as they pertain to hypokinetic dysarthria. Altered intensity feedback (AIF) is a distinct paradigm that involves the presentation of one’s own speech via headphones for the duration of the utterance (Senthinathan et al., 2021). This type of manipulation causes the participant to hear their speech at an altered (increased or decreased) intensity than is actually produced. This results in a neurologically healthy speaker adjusting their intensity to speak at a quieter loudness when hearing increased intensity feedback, as a presumed compensatory measure (Ho et al., 1999; Lane et al., 1969; Lane et al., 1961; Siegel and Pick, 1974; Senthinathan et al., 2021). Loudness perception data during AIF may provide insight into the degree of perceived congruence with speech intensity production. Evidence on loudness perception deficits in IWPD is unclear with some studies showing overestimations (Ho et al., 2000), some showing no group differences (Dromey and Adams, 2000), and some showing abnormal perception when comparing self-generated speech to externally generated speech (Clark et al., 2014; Ho et al., 1999; De Keyser et al., 2016). To our knowledge, the current study presents the first data examining loudness perception in the context of direct manipulation of auditory feedback.

Studies suggest multiple possible auditory-speech motor pathways including transmission of information through subcortical structures that may be implicated in IWPD-related hypokinetic dysarthria such as pontine nuclei and cerebellum (Glickstein, 1997), putamen, globus pallidus, thalamus (Alexander and Crutcher, 1990; Yeterian and Pandya, 1998), and what is known as the dorsal auditory stream involving the posterior superior temporal gyrus (pSTG) and the superior parietal temporal area (Buchsbaum et al., 2001; Hickok et al., 2003; Zheng et al., 2010). As the dorsal auditory stream is thought to be involved in feedback processing related to discrete speech production-related perceptual judgments (Baker et al., 1981; Miceli et al., 1980), it is possible that the current study is testing the integrity of this system in IWPD. According to the DIVA neurocomputational model of speech production, the auditory feedback control system is responsible for detecting and correcting differences between intended and current auditory signals (Manes et al., 2024). This model posits auditory target, state, and error maps lying in the pSTG with error correction commands projecting through the right ventral premotor cortex, pons, cerebellum, and ventrolateral nucleus of the thalamus (Manes et al., 2024). As such, abnormal auditory feedback responses observed in PD (Senthinathan et al., 2021) may be attributable to either disruption of the pSTG or error correction command regions.

There is a paucity of literature examining sensorimotor integration for speech production in the context of direct auditory feedback manipulation. The current study aimed to (a) examine IWPD’s speech intensity response to AIF in the context of a magnitude production task where deliberate self-monitoring and self-estimations are required to make successive increases to speech loudness, (b) examine loudness perception ratings in the context of AIF, and (c) examine speech intensity and loudness perception in the absence of auditory feedback (complete masking noise). It is hypothesized that IWPD will display an overreliance on sensory auditory feedback when making speech intensity changes and loudness perception ratings, and have exaggerated ratings when speaking in complete masking noise.

## Materials and methods

### Participants

Twenty-seven IWPD were recruited with 26 completing the full study protocol (19 men and 7 women, 69.38 ± 6.38 years old). Twenty-six neurologically healthy control participants were recruited with 24 included in the results (8 men and 16 women, 73.29 ± 5.98 years; 1 excluded for not meeting exclusion criteria and 1 due to technological issues with data processing). Control and PD groups were similar in age, *t*(48) = −1.517, *p* = 0.136. Participants with PD were recruited from patients seen by a movement disorder neurologist (M.J.) and were diagnosed as having idiopathic PD (with no concomitant neurological diagnosis) and some degree of hypophonia. To be included, all participants were required to pass a binaural hearing screen with thresholds of 40 dB HL at 0.25, 0.5, 1, and 2 kHz frequencies. Exclusion criteria for all participants included speech-language impairments besides those resulting from a diagnosis of PD, or cognitive impairment (assessed using the Montréal Cognitive Assessment score >22 required for inclusion; Nasreddine et al., 2005). Participants with PD were stabilized on their antiparkinsonian medication and were tested approximately 1 h after taking their regularly scheduled dose. The mean disease duration was 8.08 ± 5.09 years and mean Unified Parkinson’s Disease Rating Scale Part III (Goetz et al., 2008) score was 24.02 ± 7.60. All participants signed a consent form, and the research protocol was approved by the Human Subjects Research Ethics Board (Western University Ethics No. 109016). Demographic information for participants with PD is reported in Table 1.

**TABLE 1:** Parkinson’s disease patient demographic information.

Participant	Gender	Age	PD duration	Hypophonia severity	UPDRS III
PD 01	F	68	7	Mild	18
PD 02	M	71	13	Moderate	NA
PD 03	M	78	NA	Moderate	NA
PD 04	M	69	6	Moderate	36
PD 05	M	80	14	Moderate	35
PD 06	M	69	12	Mild	25
PD 07	M	75	4	Moderate	NA
PD 08	F	56	3	Moderate	NA
PD 09	M	66	10	Mild	19
PD 10	M	83	9	Moderate	NA
PD 11	M	68	3.5	Mild	11
PD 12	M	70	13	Mild	21
PD 13	M	71	5	Mod–severe	34
PD 14	M	74	2	Mild–mod	27
PD 15	M	69	10	Mild	17
PD 17	M	74	2.5	Mild	20
PD 18	M	63	6	Mild	35.5
PD 19	M	78	3	Mild	26
PD 20	M	73	7	Mild	25.5
PD 21	M	63	7	Moderate	25.5
PD 22	F	73	25	Mild	32
PD 23	F	74	11	Mild	17
PD 24	M	72	8	Moderate	30
PD 25	F	54	5	Mild	20
PD 26	F	68	4	Moderate	13
PD 27	F	64	12	Mild	17

PD, Parkinson’s disease; Hypophonia severity, as rated by experimenter; UPDRS III, Unified Parkinson’s Disease Rating Scale (Part III: Motor Examination); NA, Data not available.

### Procedure

This study was part of a larger experimental procedure that included additional speech tasks and conditions. See Senthinathan et al. (2021) for detailed apparatus, schematic of experimental setup, and calibration procedures. All participants were seated in an audiometric booth for the duration of the study. Participants were provided a set of audiometric headphones (Telephonics 51OCO17-1) and headset microphone (AKG C520) attached to a preamplifier (M-Audio preamp USB), an audiometer (GSI-10, model 1710), and a desktop computer. The microphone was placed 6 cm from the midline of the participant’s mouth. The speech task analyzed for the current study included a sentence reading (standard sentence that includes a variety of consonant and vowel sounds; useful in the acoustic analysis of PD speech “She saw patty buy two poppies”; Abeyesekera et al., 2019; Knowles et al., 2018) read in the context of a Magnitude Production Task (MP task); at a habitual speech loudness, 2× louder, 4× louder, and maximum loudness. The MP task is a frequently used method of evaluating autophonic judgment (self-perceived loudness). Using this method, the participant initially produces a spoken-stimulus and this production is assigned a value that serves as an anchor or modulus for all subsequent productions (i.e., normal loudness). The participant is then asked to produce utterances that are ratios of the initial, anchor production (i.e., 2× louder, 4× louder, etc.). This approach is systematic in its method and is based on previous psychometric research (Lane et al., 1961). The MP task requires a scaling of speech intensity and therefore deliberate monitoring of speech production intensity levels via sensory mechanisms. In other words, the MP task involves the relationship between a speaker’s perception of their speech loudness and the actual speech intensity produced.

During this task the audiometer was used to alter the intensity of the participant’s speech, who were blind to the conditions and untrained so as to capture spontaneous productions rather than possible learning effects. The AIF conditions included two repetitions of the following seven AIF conditions: 5-, 10-, and 15-dB reductions in the feedback intensity; 5-, 10-, and 15-dB increases; and 0 dB or no alteration in the feedback intensity. To minimize possible acclimation to AIF, conditions were randomized and altered feedback was turned off when participants were provided instructions for each MP task level. These conditions were completed in no noise and 100 dB of multitalker background noise (four-talker Audiotec recording played through the same audiometric headphones; total exposure duration was below OSHA noise standards), the latter in which AIF was not utilized as the noise completely masked auditory perception. Unvoiced segments or pauses (>250 ms) were selectively removed using Praat and the root-mean-square intensity contour method was used to obtain the average intensity for each utterance (Rabiner and Schafer, 1978). Participants were asked to rate the loudness of their speech during three of the seven AIF conditions (no feedback, 10 dB reduction, and 10 dB increase), in both the no noise and in the complete masking noise condition. Self-report loudness ratings were obtained by having the participants place a dash along a line on a visual analog scale (endpoints labeled low loudness and high loudness).

## Results

### Speech perception in no noise

**FIGURE 1:**
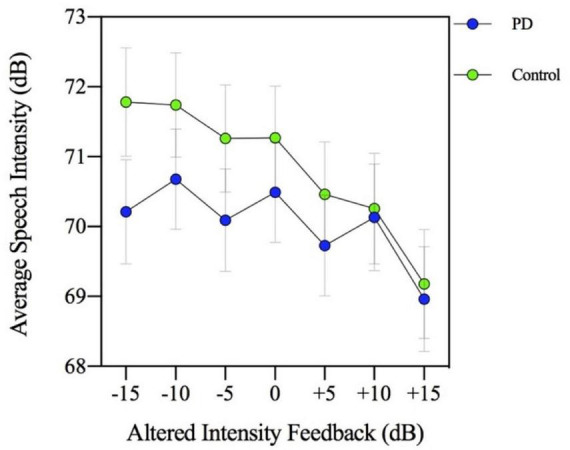
Mean speech intensity (dB) for participants with Parkinson’s disease (PD) and healthy controls. Speech intensity was measured in seven altered intensity feedback conditions (−15, −10, −5, 0, +5, +10, and +15 dB) during a magnitude production task (habitual, 2×, 4×, and max loudness).

The results of a two-way (group by MP task levels) ANOVA indicated that there was a significant main effect of the MP task [*F*(3,144) = 330.395, *p* = 0.000] with speech intensity produced by participants increasing with each successive magnitude production level (*p* = 0.000). Although the main effect of group [*F*(1,46) = 0.591, *p* = 0.446] and the group by MP task interaction [*F*(3,144) = 2.400, *p* = 0.070] were not significant, the two-way interaction involving group by AIF conditions was significant [*F*(6,288) = 9.207, *p* = 0.000]. As depicted in Figure 1, this interaction confirms that the PD and HC groups showed different responses (reduced slope by the PD group) to the AIF conditions in the context of the four MP tasks. The reduced slope response to AIF conditions compared to controls is consistent with previous work that found a reduced auditory feedback response in the PD group during other speech tasks such as reading and conversation (Senthinathan et al., 2021).

Given there was an effect of the MP task on speech, a three-way ANOVA was conducted to examine the potential modulating effect of this MP task on the AIF conditions in the two groups. This analysis examines whether there was a significant speech intensity difference based on different combinations of MP task levels, AIF condition levels, and across the two groups. The three-way ANOVA results indicate the group by AIF task by MP task interaction only approached significance [*F*(18,864) = 1.495, *p* = 0.084], suggesting that the different MP levels (2× louder, 4× louder, and max loudness) did not have a modulating effect on the AIF conditions in the PD and HC groups.

**FIGURE 2:**
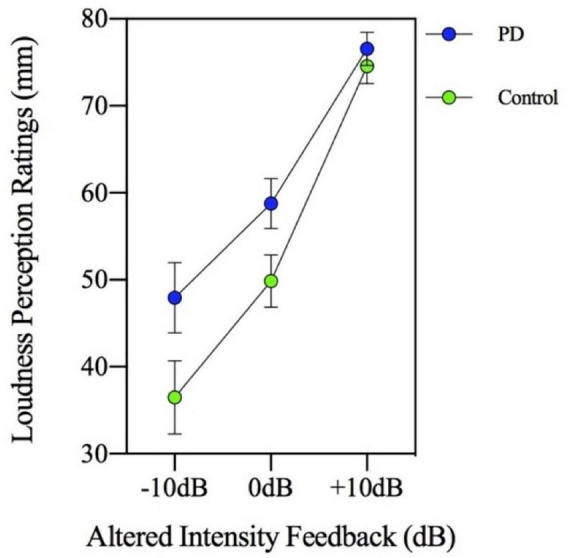
Mean loudness perception ratings measured in millimeters (mm) on a visual analogue scale for participants with Parkinson’s disease (PD) and healthy controls. Ratings were made in three altered intensity feedback conditions (−10, 0, and +10 dB) during a magnitude production task (habitual, 2×, 4×, and max loudness).

Loudness perception ratings were obtained during three of the seven AIF levels (−10, 0, and +10 dB) of the MP task. Measurement of these ratings was collected in millimeters (mm) and means were calculated for each group. A significant main effect of the MP task [*F*(3,144) = 48.002, *p* = 0.000] and a main effect of group was found [*F*(1,48) = 4.665, *p* = 0.036]. As Figure 2 suggests, the loudness perception ratings by participants increased with each successive magnitude production level (*p* < 0.001) and interestingly, the PD group was observed to have higher self-loudness ratings (*M* = 61.09; SD = 16.93) compared to the control group (*M* = 53.62; SD = 17.62). This higher self-loudness value is contrary to the lower speech intensity values that were found in the MP task.

**FIGURE 3:**
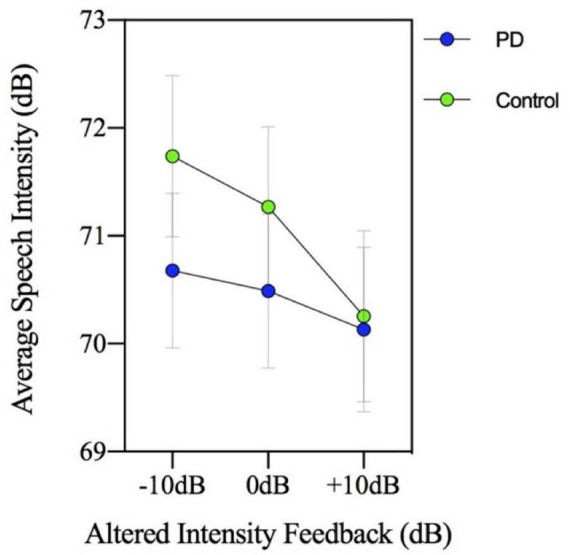
Mean speech intensity (dB) for participants with Parkinson’s disease (PD) and healthy controls. The figure shows a portion of the results previously presented in Figure 1 and is being re-presented to show three of the seven altered intensity feedback conditions for the purpose of contrasting the loudness perception ratings (Figure 2) with the speech production data. Loudness perception ratings were only collected during −10, 0, and +10 dB altered intensity feedback conditions.

The group by MP task interaction was not significant [*F*(3,144) = 0.717, *p* = 0.543] indicating there was no significant group difference in loudness perception ratings across the MP task levels in the context of AIF. In addition, the two-way interaction involving group by AIF conditions was not significant [*F*(2,96) = 2.039, *p* = 0.136]. Therefore, the PD and control groups had similar loudness perception ratings across the different AIF conditions, despite consistently showing significantly different speech intensity responses. Figure 2 (loudness perception ratings) and Figure 3 (speech intensity responses) highlight the distinction between the speech intensity responses and the loudness perception ratings to AIF in the two groups. Figure 3 contains a portion of the results previously presented in Figure 1 and is being re-presented for the purpose of contrasting the loudness perception ratings (Figure 2) which were gathered only during the −10, 0, and +10 dB AIF conditions, with the speech production data.

**FIGURE 4:**
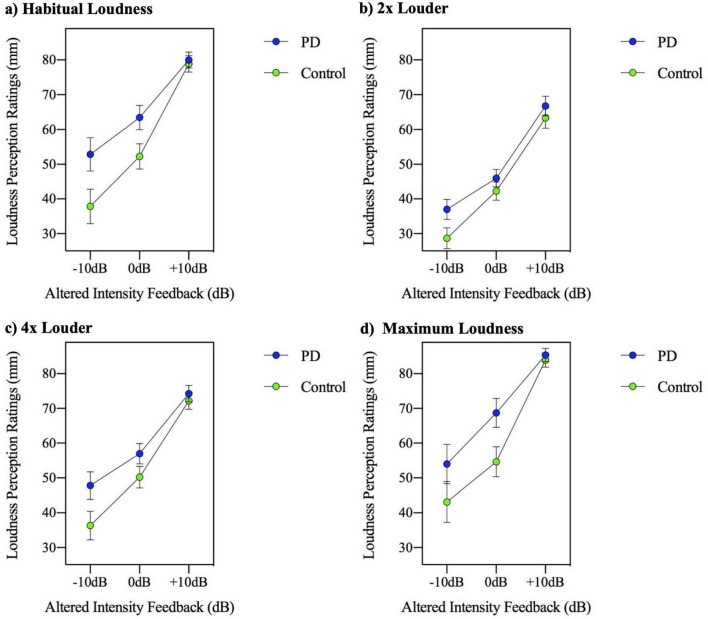
Mean loudness perception ratings measured in millimeters (mm) on a visual analogue scale, for participants with Parkinson’s disease (PD) and healthy controls across three altered intensity feedback conditions in the reading at **(a)** habitual loudness, **(b)** 2× louder, **(c)** 4× louder, and **(d)** maximum loudness.

A three-way (group by AIF feedback condition by MP level) repeated measures ANOVA for the dependent measure of loudness perception rating was performed. The three-way ANOVA results indicate the group by AIF task by MP task interaction was significant [*F*(6,288) = 2.288, *p* = 0.036], suggesting that although the MP levels did not have a modulating effect on the AIF conditions in the two groups for the dependent variable of speech intensity, the MP levels did have a modulating effect for the dependent variable of loudness perception. This three-way interaction is depicted in Figure 4. It appears this significant interaction is a result of the loudness perception in the 4× loudness (Figure 4c) and maximum loudness (Figure 4d) MP conditions. It appears that the control group produced a steeper slope of loudness ratings across AIF levels compared to the relatively consistent flatter slope of loudness ratings by the PD group.

Overall, the analysis of speech in no noise suggests that while speech intensity increases with each MP task level, there were group differences in this response. The PD group displayed a reduced slope of intensity across the MP task. The loudness perception ratings increased across each successively louder MP task production, however the analysis revealed that while the PD group appeared to have overall increased self-loudness perception ratings, this was not statistically significantly different. Therefore, in the context of AIF and the MP task, ratings were the same across both groups despite differing speech intensity productions. Interaction analysis shows that the 4× louder and max loudness MP task levels may be driving the steeper slope of loudness perception ratings in the control group compared to the consistently flatter slope of the PD group.

### Speech perception in complete masking noise

The descriptive statistics related to the masking noise conditions (no noise and 100 dB masking noise) for both the PD and control groups are shown in Table 2. The results of a two-way (group by masking noise condition) ANOVA indicated that there was a significant main effect of masking noise [*F*(1,48) = 21.208, *p* = 0.000]. *Post hoc* analysis of simple main effects revealed that the 100 dB masking noise condition (*M* = 74.54; SD = 3.27) was associated with higher speech intensity relative to the no noise condition (*M* = 72.11; SD = 4.41) (*p* = 0.000). The main effect of group [*F*(1,48) = 2.071, *p* = 0.157] and group by noise condition interaction were not significant [*F*(1,48) = 0.155, *p* = 0.695, respectively].

**TABLE 2:** Descriptive statistics of speech intensity (measures in dB) means and standard deviations related to the masking noise conditions obtained for the PD (*n* = 26) and HC (*n* = 24) groups in the MP task.

Masking noise condition	PD		HC	
	**Mean (dB)**	**SD**	**Mean (dB)**	**SD**
No noise	71.52	4.76	72.70	3.97
100 dB noise	73.75	2.74	75.34	3.76

**FIGURE 5:**
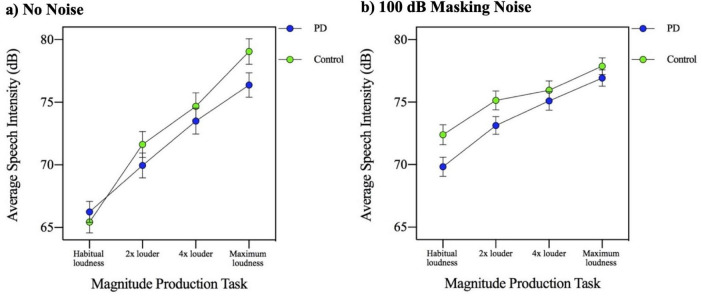
Mean speech intensity (dB) for participants with Parkinson’s disease (PD) and healthy controls across four magnitude production task levels (habitual, 2×, 4×, and maximum loudness) in the context of **(a)** no background noise and **(b)** 100 dB masking noise.

In order to examine the effect of complete masking noise on the four MP task conditions, a three-way ANOVA involving masking noise, MP task and group was used. The results of the three-way (group by masking noise by MP task) ANOVA indicated that there was a significant main effect of MP task [*F*(3,144) = 260.754, *p* = 0.000]. *Post hoc* analysis of simple main effects related to the 4 MP tasks conditions (habitual loudness, 2× louder, 4× louder, and maximum loudness), revealed that speech intensity increased with each successive MP loudness task. The group by MP task interaction was not significant [*F*(3,144) = 1.148, *p* = 0.332]. The results of the three-way ANOVA also indicated that there was a significant three-way interaction, involving group, masking noise condition and MP task [*F*(3,144) = 6.617, *p* = 0.000]. To interpret this three-way interaction, a separate plot of the two-way MP task by group interaction was created for each of the two masking noise conditions (no noise and 100 dB masking noise). These two plots are shown in Figures 5a, b. Visual inspection of these two figures indicates that while in the no noise condition the group difference is most apparent in the higher MP task conditions (4× louder and maximum loudness), in the complete masking noise, the group difference is most apparent in the lower MP task conditions (habitual loudness and 2× louder).

Self-loudness perception ratings were also obtained during the MP task in the context of complete masking noise (100 dB background noise). In order to examine the effects of complete masking noise on loudness perception ratings in PD and HC groups, a two-way ANOVA involving noise conditions (no noise and 100 dB masking noise) and group factors was used. The results of the two-way (group by noise conditions) repeated measures ANOVA for loudness perception indicated the main effect of noise conditions was not significant [*F*(1,48) = 2.618, *p* = 0.112]. This result suggests that the participants rated their speech loudness as similar whether in no noise or in complete masking noise despite producing a significantly increased speech intensity in the complete masking noise condition compared to the no noise condition. The main effect of group [*F*(1,48) = 2.089, *p* = 0.155] and the group by noise condition interaction [*F*(1,48) = 3.298, *p* = 0.076] were not significant. It should be noted that although this interaction was not statistically significant, the control group rated their speech as louder in the complete masking noise whereas the PD group did not (see Table 3).

**TABLE 3:** Descriptive statistics of loudness perception rating (measured in mm) means, and standard deviations related to the masking noise conditions obtained for the PD (*n* = 26) and HC (*n* = 24) groups in the context of the MP task (habitual loudness, 2× louder, 4× louder, and maximum loudness).

Masking noise condition	PD		HC	
	**Mean (mm)**	**SD**	**Mean (mm)**	**SD**
No noise	60.89	14.36	50.15	16.42
100 dB noise	60.24	17.14	61.41	17.82

A three-way ANOVA (MP task by noise conditions by group) was used to examine the loudness perception ratings during the MP tasks when combined with the masking noise conditions. The main effect of MP task was significant [*F*(3,144) = 92.760, *p* = 0.000] with loudness perception ratings by participants increasing with each successive magnitude production level (*p* < 0.000). This is consistent with the speech intensity levels that were being produced. The three-way ANOVA results indicate the group by noise conditions by MP task interaction was not significant [*F*(3,144) = 2.364, *p* = 0.074].

Overall, the analysis suggests that when speaking in the context of complete masking noise, speech intensity increased when speaking in noise compared to no noise and increased across the MP task levels. This was the same for both control and PD participants. While the MP task group differences may have been driven by the 4× louder and max loudness requests in no noise, the group differences may be driven by the habitual and 2× louder requests in complete masking noise. Interestingly, while self-loudness perception ratings increased across successively louder MP task levels, no group differences were observed.

## Discussion

The MP task is inherently complex, as it requires the speaker to perceive the loudness of their voice, estimate a comparatively higher level of self-loudness, and accurately perform the motor output to achieve the intended loudness. This sensorimotor integration task (requiring the integration of auditory information and motor pathways for speech production) therefore involves deliberate self-estimation and self-monitoring of speech production with a greater degree of focus on internal targets relative to other speech tasks (i.e., conversation and imitation tasks) and the MP task may require less external guidance or focus than other speech tasks such as imitation. Overall, all participants in the current study were observed to successfully complete the task and scale the intensity of their speech across MP task conditions. The current study is consistent with work by Dromey and Adams (2000) who did not find a significant difference between PD and control participants. In contrast, a previous study by Clark et al. (2014) found a flatter slope of the loudness function in their PD participants. However, Clark et al. (2014) examined a wider range of MP task conditions (i.e., two additional soft conditions; 2× and 4× softer), and the flatter slope found in their study may be attributed to these additional conditions. It is worth noting, however, that although the difference between groups did not reach significance in the present study, the PD participants were observed to produce a slightly flatter slope of the MP response than the controls. If PD speakers have a particular deficit in the processing of external feedback for motor control (i.e., excessive inhibition), perhaps the highly internal focus of the MP task is why they are generally more successful in achieving a similar MP function to controls. In contrast, the overall gain setting was abnormal in the PD group (overall reduced loudness compared to controls), but this initial gain setting may be less reliant on internal targets. The slightly reduced slope produced by the PD group in the AIF conditions cannot be explained by a reduced capacity. In Figure 1 a restricted slope of the PD group (intensity range is ∼69–70.7 dB) is observed. However, in Figure 5, when producing speech in complete masking noise, the PD group producing intensity that is >75 dB.

All participants rated their speech as successively louder with each successive MP condition. Of interest, the PD group was observed to rate the loudness of their speech as being louder compared to the control group despite the PD group producing reduced speech intensity. Consistent with previous studies of loudness perception in PD, the current study found that individuals with PD have an inaccurate perception of their self-generated speech loudness (Clark et al., 2014; Ho et al., 1999; Ho et al., 2000; De Keyser et al., 2016; Kwan and Whitehill, 2011) and overestimate their loudness. A unique contribution of this work is that although the PD group produced a flatter slope of the function in the MP task, they nevertheless rated the loudness of the speech similarly to how control participants rated their loudness. These results suggest that individuals with PD may have an under reliance on auditory sensory feedback such that their productions and perception of those productions may not rely on the sensory feedback being received. Some auditory cortical areas have been observed to increase in activity, known as a speech perturbation response enhancement (SPRE), with altered auditory feedback (Behroozmand et al., 2009; Zheng et al., 2010). It is also possible that abnormal processing of auditory sensory feedback is occurring in this region for individuals with PD. The loudness perception ratings pose possible challenges particularly related to motor control as well as the cognitive processes required to complete the task (i.e., loudness perception ratings require an individual to remember the intensity that was produced and convert that to a scaled response). The cognition screen was used to help mitigate any possible processing challenges. The participants were monitored closely during the loudness perception task to ensure that motor movements were not confounding the data collection. Participants would be asked to confirm their ratings especially if motor concerns were apparent. However, because participants were “on” medication, this was not a notable issue in the current study. Debriefing following the ratings did not yield any concerns related to the physical nature of the loudness perception ratings. It should be noted that although a “loudness” perception rating was required by participants, it is possible that participants were using other or multiple sensory feedback processes (proprioception, effort, level of fatigue, etc.) to make these determinations and the relative weighting of these processes may be different during AIF in general, in PD, or in controls. This means that ratings of loudness may in fact be representing ratings of effort and that a perceived increase in effort is being experienced in PD patients. By directly manipulating the loudness level of self-produced speech in the current study, we attempted to control for alternative sensory processes, however it is not possible to conclude that auditory mechanisms were being used exclusively in the no noise condition. In addition, it is important to note the lack of sex matching across groups and possible lingering effects of AIF are limitations of the current study and may have implications for speech production and perception results.

Speaking-induced suppression has been observed in the auditory cortex during self-produced speech such that the activity in the auditory cortex is reduced compared to when externally produced speech is played to a participant (Curio et al., 2000; Greenlee et al., 2011; Houde and Jordan, 2002). However, it has been suggested that although the auditory cortex functions to suppress function with expected auditory feedback, once there is a mismatch with this expectation, the auditory cortex is once again primed. (Behroozmand et al., 2009; Chang et al., 2013; Eliades and Wang, 2008; Greenlee et al., 2011; Houde and Jordan, 2002). Studies have found the superior temporal gyrus (STG) (Fu et al., 2006; Tourville et al., 2008; Parkinson et al., 2012; Zheng et al., 2010), and ventral supramarginal gyrus (vSMG) (Tourville et al., 2008; Toyomura et al., 2007) to be active during altered auditory feedback. Tourville et al. (2008) also found activation in superior cerebellum, ventral thalamus, and anterior striatum, with the additional regions of bilateral superior cerebellar cortex, medial parietal-occipital cortex, and right lateralized inferior cerebellar cortex active during altered pitch feedback. Thus, complex sensory-motor networks are involved in speech production with altered auditory feedback and sensory activation of motor control areas may be responsible for the compensation of erred feedback. Further work in the area of speech intensity regulation is needed.

The complete masking condition required participants to create an internal representation and scaling of the production of speech intensity across different loudness levels, in the absence of auditory feedback. This condition may provide insight into the relative importance of external auditory feedback and the degree of internal focus during this task. In the context of speech produced in no noise, control speakers may have a primarily internal focus, however there is a degree of feedback monitoring that occurs and is required in order to regulate their loudness (Senthinathan and Adams, 2020; Senthinathan et al., 2021) and without this feedback (i.e., complete masking), the appropriate scaling of loudness across the MP conditions is disrupted. In contrast, the current results suggest that the PD group do not utilize auditory feedback when completing a MP task and therefore the complete masking of auditory feedback had no effect on their performance of the MP task. Some studies have found the mid-to-posterior STG to be more active when auditory feedback was completely masked (Christoffels et al., 2007; van de Ven et al., 2009), highlighting the importance of the STG in auditory processing of self-generated speech. A previous study by Vikene et al. (2019) found that the STG in PD patients displayed increased activity during an auditory omission detection task suggesting that regulation of self-produced speech may have a distinct pattern of activation in the brain.

To our knowledge, this is the first study to examine loudness perception ratings in complete masking noise. Since no auditory feedback was available during this task, the participants were to use any strategy to make their loudness ratings. If a participant inquired about how to rate their loudness, they were encouraged to use alternate methods such as ratings based on “how it feels” or “how much effort.” Although participants accurately rated their loudness as successively louder with each MP condition, they were observed to have overall similar ratings of loudness whether in no noise or in complete masking noise. This did not align with the increased intensity that was produced in the complete masking noise condition and suggests that it is difficult to make loudness perception ratings when auditory feedback is completely blocked.

Interestingly, the PD and control group ratings were not statistically different, however a trend was observed in the data such that the control group rated their speech as louder in complete masking noise (consistent with the increase in intensity). The PD group was not observed to perceive an intensity increase when speaking in complete masking noise. This contrasts with the PDs overestimations of their loudness in the no noise condition. As previously noted, it is possible that when making loudness perception judgments in the no noise condition, participants were actually using other sensory processes such as perceived effort. In the complete masking noise condition, when no auditory feedback is possible, the study attempts to uncover these other possible processes. In this condition, PD participants increased their speech intensity, which would presumably lead to a further increase in perceived effort. This is because in order to produce an increase in speech intensity, increased muscular effort is required at both the laryngeal and respiratory system levels (van den Berg, 1958). However, we do not see a difference in loudness perception ratings in this complete masking noise condition compared to no noise. As such, the current study findings suggest that somatosensation or sense of effort is modulated differently by sensory feedback in the PD group.

## Data Availability

The raw data supporting the conclusions of this article will be made available by the authors, without undue reservation.
